# Identification of a gene network driving the attenuated response to lipopolysaccharide of monocytes from hypertensive coronary artery disease patients

**DOI:** 10.3389/fimmu.2024.1286382

**Published:** 2024-02-12

**Authors:** Chang Lu, Marjo M. P. C. Donners, Julius B. J. de Baaij, Han Jin, Jeroen J. T. Otten, Marco Manca, Anton Jan van Zonneveld, J. Wouter Jukema, Adriaan Kraaijeveld, Johan Kuiper, Gerard Pasterkamp, Barend Mees, Judith C. Sluimer, Rachel Cavill, Joël M. H. Karel, Pieter Goossens, Erik A. L. Biessen

**Affiliations:** ^1^ Department of Pathology, Cardiovascular Research Institute Maastricht (CARIM), Maastricht University Medical Center, Maastricht, Netherlands; ^2^ Institute for Computational Biomedicine, Faculty of Medicine, Heidelberg University and Heidelberg University Hospital, Heidelberg, Germany; ^3^ Science for Life Laboratory, KTH - Royal Institute of Technology, Stockholm, Sweden; ^4^ SCimPulse Foundation, Geleen, Netherlands; ^5^ Department of Internal Medicine (Nephrology), Leiden University Medical Center, Leiden, Netherlands; ^6^ Department of Cardiology, Leiden University Medical Center, Leiden, Netherlands; ^7^ Netherlands Heart Institute, Utrecht, Netherlands; ^8^ Department of Cardiology, University Medical Center Utrecht, Utrecht, Netherlands; ^9^ Division of BioTherapeutics, Leiden Academic Centre for Drug Research, Leiden University, Leiden, Netherlands; ^10^ Circulatory Health Research Center, University Medical Center Utrecht, Utrecht, Netherlands; ^11^ Department of Vascular Surgery, Maastricht University Medical Center, Maastricht, Netherlands; ^12^ Centre for Cardiovascular Science (CVS), University of Edinburgh, Edinburgh, United Kingdom; ^13^ Department of Advanced Computing Sciences, Maastricht University, Maastricht, Netherlands; ^14^ Institute for Molecular Cardiovascular Research, Klinikum RWTH Aachen, Aachen, Germany

**Keywords:** coronary artery disease, hypertension, circulating monocytes, inflammatory responses, gene regulatory network

## Abstract

**Introduction:**

The impact of cardiovascular disease (CVD) risk factors, encompassing various biological determinants and unhealthy lifestyles, on the functional dynamics of circulating monocytes—a pivotal cell type in CVD pathophysiology remains elusive. In this study, we aimed to elucidate the influence of CVD risk factors on monocyte transcriptional responses to an infectious stimulus.

**Methods:**

We conducted a comparative analysis of monocyte gene expression profiles from the CTMM – CIRCULATING CELLS Cohort of coronary artery disease (CAD) patients, at baseline and after lipopolysaccharide (LPS) stimulation. Gene co-expression analysis was used to identify gene modules and their correlations with CVD risk factors, while pivotal transcription factors controlling the hub genes in these modules were identified by regulatory network analyses. The identified gene module was subjected to a drug repurposing screen, utilizing the LINCS L1000 database.

**Results:**

Monocyte responsiveness to LPS showed a highly significant, negative correlation with blood pressure levels (ρ< -0.4; P<10^-80^). We identified a ZNF12/ZBTB43-driven gene module closely linked to diastolic blood pressure, suggesting that monocyte responses to infectious stimuli, such as LPS, are attenuated in CAD patients with elevated diastolic blood pressure. This attenuation appears associated with a dampening of the LPS-induced suppression of oxidative phosphorylation. Finally, we identified the serine-threonine inhibitor MW-STK33-97 as a drug candidate capable of reversing this aberrant LPS response.

**Conclusions:**

Monocyte responses to infectious stimuli may be hampered in CAD patients with high diastolic blood pressure and this attenuated inflammatory response may be reversed by the serine-threonine inhibitor MW-STK33-97. Whether the identified gene module is a mere indicator of, or causal factor in diastolic blood pressure and the associated dampened LPS responses remains to be determined.

## 1 Introduction

Cardiovascular diseases (CVD), including ischemic heart disease and stroke are the major causes of death worldwide. Recognized factors such as gender, age, ethnicity, hyperlipidemia, diabetes, hypertension, sedentary lifestyle, and smoking have been identified as contributors to increased CVD risk, with atherosclerosis serving as its primary underlying pathology (1). Of these factors, hypertension emerges as a pervasive, modifiable risk element exacerbating atherosclerosis, particularly in the coronary and cerebral arteries (2, 3). Despite the significance of these traditional risk factors, or composites thereof (like the Framingham score), their predictive value at the individual level remains limited. Seeking more precise indicators for future CVD events, numerous biomarker discovery studies have concentrated on plasma or serum molecules, yielding modest success. The CIRCULATING CELLS study (4) was conceived to unveil and validate cell-based biomarkers for risk prognostication in patients with coronary artery disease (CAD). Among accessible cell types, blood monocytes, characterized by their ready trauma sensing capacity and adaptability, emerge as promising targets for biomarker discovery (5). Moreover, monocytes, as precursors of macrophages, are not only important for homeostatic organ control, but also important contributors to CVD progression as well as infarct repair (6–8).

Interestingly, circulating monocytes have not only been associated with CVD progression but are also impacted by CVD risk factors. For example, the number of monocytes, in particular in the CD14^++^CD16^+^ monocyte subset, is increased in patients with acute myocardial infarction (5, 9) and associated with further disease progression (10). Both aging and male gender were shown to increase monocyte numbers and pro-inflammatory cytokine production (11, 12). Smoking was also shown to affect circulating monocyte numbers (13, 14) but to decrease their chemotactic capacity (13). Interestingly, monocytes from symptomatic CVD patients are primed *in vivo* to produce more pro-inflammatory cytokines after an *ex vivo* stimulation with the bacterial endotoxin lipopolysaccharide (LPS), linking infection to CVD (15). Undoubtedly, the pro-inflammatory signaling of monocytes/macrophages through toll-like receptors, such as toll-like-receptor-4 (TLR4), triggered by bacterial endotoxins like LPS, has been implicated in the pathogenesis of atherosclerosis (16, 17). Significantly, even in seemingly healthy individuals without clinical evidence of infection, levels of endotoxin in plasma can be sufficient to induce inflammatory responses in monocytes/macrophages and were seen to be linked to an elevated risk of atherosclerosis (18).

In addition, hyperlipidemia, another prominent risk factor for CVD, is recognized for inducing monocytosis and activating monocytes (19, 20), while a Western type diet feeding or oxidized low-density lipoprotein exposure are reported to produce long-term pro-inflammatory changes in monocytes by genetic and metabolic imprinting, a process called trained immunity (21, 22). Besides hyperlipidemia, however, still little is known about the genomic imprint of other CVD risk factors in monocytes that may impact monocyte’s secondary responses to foreign substances, e.g. during bacterial infections.

Integrating transcriptomics analysis on naïve and LPS stimulated monocytes and patients’ cardiovascular risk profile of a sub cohort of the before mentioned CIRCULATING CELLS study, we here aimed to dissect the impact of CVD risk factors on monocyte phenotype, and in particular on their response to trauma and infection response.

## 2 Materials and methods

### 2.1 Human blood samples

This study enrolled 50 patients with stable angina pectoris from the Center for Translational Molecular Medicine (CTMM) – CIRCULATING CELLS Cohort (4), who presented at the Maastricht University Medical Center in The Netherlands. Upon inclusion, comprehensive case record forms were completed, capturing detailed information such as medical history, risk factors, medication usage, extent and severity of coronary artery disease, laboratory measurements, and final procedural outcomes. Coronary angiography adhered to local standards, and the anatomical severity of coronary artery disease was evaluated by calculating the SYNTAX score for each patient, as previously described (4). Blood pressure was measured at the time of inclusion using standard upper arm cuff measurements.

Exclusion criteria were active inflammatory conditions, autoimmune disease, malignancies, use of immunosuppressive drugs, and known hematological disorders. Blood of patients with suspected unstable angina and ST-elevation myocardial infarction were also not included in these 50 subjects. This study was approved by the institutional medical ethical review board of the university medical center Utrecht, the Netherlands.

### 2.2 Cell isolation and treatments

Upon inclusion, 50 blood samples were collected in ethylenediaminetetraacetic acid (EDTA) anti-coagulated vacuum tubes (Becton Dickinson, Breda, The Netherlands; 1.5 mg EDTA/ml blood) and processed according to standardized procedures to analyze leukocyte subsets. In short, blood was transferred to a 50 mL tube (Greiner) and spun for 15 minutes at 156g without brake. Subsequently, plasma was removed, Phosphate Buffered Saline (PBS) was added to a volume of 70 mL, then 35 mL PBS-diluted blood cell suspension was added on top of 15 mL Ficoll-Paque Plus (Sigma) and spun for 20 minutes at 1000g without brake. The leukocyte enriched interphase was collected and washed twice in BD Imag cell selection buffer (containing PBS 0.5% BSA, 2mM EDTA and 0.09% sodium azide) before CD14 positive monocyte isolation according to the manufacturer’s protocol (BD Bioscience). In short, cells were incubated with anti-human CD14 magnetic particles (75 µl/10^7^ cells, BD Biosciences) for 1h on ice. Magnetic selection was performed using a pre-cooled BD Imagnet kept on ice. Isolated CD14^+^ monocytes were gently frozen (200 µl cell suspension in PBS in 1 ml Freezing medium (RPMI+20% DMSO) was placed for 1h at -20°C, then 3-5 days in -80°C) and stored at -180°C until further use. Monocytes from 50 patients with stable angina were then treated with 100 ng/mL LPS (type 055:B5, Sigma) in RPMI containing 10% fetal calf serum for 15 min before RNA isolation.

### 2.3 RNA isolation and micro-array analysis

RNA isolation, quality check, and microarray analysis of the baseline and LPS-stimulated monocyte samples were done by AROS (Denmark). In brief, total RNA was isolated using Illumina TotalPrep RNA Amplification Kit (Illumina, San Diego, CA, USA) and cDNA was produced. Next, labelled cRNA was prepared and used on the array (Human HT-12 beadchips. V3.0) for hybridization. Hybridized chips were scanned by Illumina BeadStation (Illumina, Inc., San Diego, CA, U.S.A.). Raw image analysis and signal extraction was performed with Illumina BeadStudio Gene Expression software with default settings (no background subtraction) and no normalization. Data were exported as text files.

### 2.4 Data Pre-processing

Gene expression microarrays were exported to R v3.6.3 after quality control using GenomeStudio software. Variance stabilizing transformation and robust spline normalization were then performed using lumi package (23). Genes with expression significantly above the background (defined by negative control probes in GenomeStudio) were regarded as detectable genes (*P*<0.05). We excluded 5 of the 50 microarrays from the baseline group and 6 of the 50 microarrays from the LPS-stimulated group, respectively, due to too low numbers of detectable genes (<9,000). Pairing the patient IDs of the remaining 44 LPS-stimulated and the 45 baseline monocyte microarrays revealed that 39 patients contained both baseline and LPS-stimulated microarrays for subsequent pair-wise analyses. To reduce the impact of noise genes, we then filtered out genes with borderline (log_2_(Intensity) <7.5 across all patients) or low variance expression, providing a robust 7,933 gene dataset for further analysis (SD < 0.5). Therefore, matched baseline and LPS-stimulated microarrays from 39 CAD patients were used for generating the LPS-response matrix and differential gene expression analysis. The schematic diagram of the cohort build-up is shown in **Supplementary Figure 1A**. The LPS-response matrix was composed of each CVD patient’s log2 fold-change (log_2_FC) of LPS-stimulated versus baseline expression. Agglomerative hierarchical clustering of the LPS-response matrix based on Euclidean distance led to the detection of one significant outlier patient (patient ID: 31031) (**Supplementary Figure 1B**). Eventually, the LPS-response matrix including 38 patients’ log_2_FCs were used for the following differential gene expression and co-expression analysis. The summary of 14 CVD risk factor of these 38 patients is shown in **Table 1**.

**Table 1:** Demographics of the LPS sub cohort of CTMM CIRCULATING CELLS (n=38 CAD patients) and CVD risk factors, expressed as mean ± sd, count or frequencies (%).

Binary CVD risk factor	Count	Percentage
**Sex (male)**	20	52.63%
**Renal Failure (yes)**	3	7.89%
**Current Smoker (yes)**	7	18.42%
**Diabetes Mellitus (yes)**	7	18.42%
**Other CVD risk factor**	**Mean ± sd**	
**Age**	66.68 ± 8.65 years	
**BMI**	27.13 ± 4.14 kg/m^2^	
**Heart Rate**	63.29 ± 10.32 bpm	
**DBP**	74.78 ± 11.88 mmHg	
**SBP**	137.68 ± 21.35 mmHg	
**Glucose**	6.41 ± 1.15 mmol/dL	
**Triglyceride**	1.55 ± 0.8 mmol/dL	
**Creatinine**	87.45 ± 21.3 mmol/dL	
**HDL**	1.11 ± 0.3 mmol/dL	
**LDL**	2.64 ± 0.91 mmol/dL	

### 2.5 Differential gene expression analysis

To investigate the association between LPS response and CVD risk factors, we estimated the average fold changes and standard errors by limma linear model fitting (24), comparing LPS-stimulated and baseline gene expressions. P-values were adjusted by False Discovery Rate (FDR).

### 2.6 Gene co-expression network clustering analysis

Weighted Gene Co-expression Network Analysis (WGCNA) (25) was carried out on the LPS-response matrix. In brief, the adjacency matrix was built on gene co-expression similarity based on Pearson’s Correlation coefficient across genes. The soft-thresholding power of the network was set as 5, to approximate a scale-free topological network topology. As distance measure for gene clustering the dissimilarity of the topological overlap between two genes was used, based on the gene adjacency matrix [see (25)]. Hierarchical clustering (deepSplit = 3, cutHeight = 0.995, minClusterSize = 50) was performed on the topological overlap, followed by merging proximal clusters (Height < 0.2) in the hierarchy tree. Eventually, 20 gene modules (clusters) with similar LPS responses were generated, which were color coded. For each gene module, Principal Component Analysis was performed, regarding patients as samples and genes as variables. The module’s eigengene was then defined as the eigenvector of the first principal component and used for subsequent Bayesian network construction and visualization of the hierarchical tree dendrogram.

### 2.7 Gene set over-representation analysis

The enrichment score of Gene Ontology and pathway over-representation analyses were calculated using runGSAhyper in the piano R-package (26). The Gene Ontology and pathway databases for enrichment analyses were downloaded from Human Molecular Signatures Database (MSigDB) (http://www.gsea-msigdb.org/gsea/msigdb/collections.jsp). Fisher’s exact test was applied on up and down-regulated genes separately, with the total number of background genes being 7,933. The p-values were adjusted for multiple comparisons by FDR.

### 2.8 Bayesian network analysis

Bayesian network inference was performed on blood pressure levels and the eigengenes of 10 gene modules that have at least one enriched Gene Ontology term, using the R package bnlearn (v4.5) (27), to infer causal relationships between the biological processes represented by the gene modules and diastolic blood pressure (DBP). To bolster the resilience of the network, we employed a bootstrapping approach to learn network structures. Initially, we randomly selected 20 genes from each module and computed their average expression, generating a sample-module matrix. Subsequently, Bayesian network structures were learned using hill climbing. This bootstrapping procedure was iterated 1,000 times, resulting in 1,000 Bayesian network structures. Confidence (strength) for each network edge was then determined based on the frequency of its occurrence across all networks (28). The confidence for the direction of an edge is calculated as the probability of that direction occurring divided by the probability of the edge occurring in all networks (29). The consensus network was established by selecting the direction present in at least 50% of cases, with an edge strength higher than 0.56 (the elbow point of the cumulative distribution function of edge strengths). Ultimately, a directed acyclic Bayesian network was constructed which comprised 10 nodes and 11 edges, representing the relationships between modules and DBP.

### 2.9 Gene regulatory network reconstruction

An LPS Gene regulatory network was generated from an LPS response matrix including 38 samples and 7,933 genes, using algorithm for the reconstruction of accurate cellular networks (ARACNe) (30, 31) [ARACNe-AP v1.4 (31)], an information theoretic-based network reconstruction method. We first built a gene co-expression network based on mutual information (MI) between genes and transcription factors. After estimating the significance threshold of MI values, we reconstructed 100 MI networks by bootstrapping of the LPS response matrix, and then removed the non-statistically significant links with MI <0.5. The indirect interactions were then deleted by applying a Data Processing Inequality tolerance filter (30), to recover transcriptional interactions with high confidence. A consolidated gene regulatory network including 31,884 genes was inferred by calculating the Bonferroni-corrected significance of the relative appearance of each edge in the bootstraps based on a Poisson distribution, and only keeping the significant edges (Padj < 0.05). In total of 1,614 human transcription factors with known Entrez IDs as regulators (downloaded from http://humantfs.ccbr.utoronto.ca/).

### 2.10 Transcription factor activity inference

We used an analytic Rank-based Enrichment Analysis method called VIPER (32) to infer each patient’s LPS-dependent transcription factor activities, based on the reconstructed gene regulatory network and the LPS-response matrix. VIPER predicts a transcription factor’s activity by comparing the order-ranked gene expression signature and the correlation of the expressions between this transcription factor and its targets. These procedures converted the gene-sample matrix to a transcription-factor-sample activity matrix including 439 transcription factors and 38 patients. The p-values of transcription factor’s enrichment scores were corrected for multiple comparisons by FDR.

### 2.11 Co-expression network and gene regulatory network of module salmon

By applying a cut-off of 0.4 for network edge weights (based on weighted correlations defined by WGCNA), the salmon module was extracted from the entire WGCNA co-expression network, resulting in a subnetwork comprising 155 gene nodes. The hub genes of this subnetwork were identified by assessing five network centrality measures (i.e. degree, betweenness, closeness, eigenvector and page rank centrality) for all nodes from module salmon using igraph R package (33), ranking centralities for each node, and integrating the resulting rank lists using Robust Rank Aggregation (34). This tool compares each gene’s position on the list to the null hypothesis of random orders and estimating their significance score. Module genes with a Bonferroni corrected p-value for the aggregate gene rank lower than 0,05 were considered to be hub genes (40 in total).

For the gene regulatory network, we selected all genes in module salmon, the directly connected transcription factors thereof, as well as all edges between these two entities, resulting in a total of 218 gene nodes in the salmon sub gene regulatory network. Both the WGCNA network and the gene regulatory network for the module salmon were visualized by Cytoscape (35). The weight of edges in gene regulatory network were visualized as the Mode of Regulation (MoR) from (32), which was determined based on the Spearman correlation coefficient between a transcription factor and its target expression, indicating the degree of suppression (MoR <0) and activation (MoR > 0) of target genes by the transcription factor. Correlation between a transcription factor and the DBP level was computed as the Pearson correlation coefficient between a transcription factor’s activity and the DBP level across all patients.

### 2.12 Drug repurposing

We performed drug screening based on the LINCS L1000 database to discover drugs able to reverse the high blood pressure associated changes in the LPS response of module salmon genes (36). Only datasets on relevant myeloid cell lines (i.e., HL60, THP1, NOMO1, SKM1, PL21, U266, HS27A) and genes accessible in the LINCS database were included in this study. The connectivity scores based on the weighted bi-directional Kolmogorov-Smirnov (K-S) enrichment statistic between changes of drug-induced gene expression in the LINCS L1000 database and hub genes in module salmon were calculated as described (36). A positive score indicates there is a similarity between a drug-induced signature in the L1000 reference database and LPS response of hub genes in module salmon, while a negative score indicates that these two signatures are opposing. Since all hub genes in module salmon were downregulated by LPS and their expression increased with the growth of DBP, any drug with a positive connectivity score indicates that it may be able to rescue the dampened LPS response in DBP.

### 2.13 Statistical analysis

For all 7933 genes, we calculated the Pearson’s Correlation coefficients between 14 CVD risk factors and log_2_FC of each gene across all patients from the LPS response matrix. P-values were computed using the student t-test. The correlation between gene modules and risk factors, and among risk factors were also measured by Pearson’s Correlation Coefficients with p-values. Principal Component Analysis was performed on the top 25% of the highest expressed genes from the baseline and LPS-stimulated expression matrices (**Supplementary Figure 2**). The association of gene expression projections on the top 2 principal components (PC1 and PC2) and risk factors were calculated by Pearson’s Correlation coefficients. In addition, multiple linear regression models were developed between the eigengenes in each module and DBP with diabetes, glucose, triglyceride (TG), high-density lipoprotein (HDL), and low-density lipoprotein (LDL) as covariates using limma (24), and the p-value of each variable was calculated based on the moderated t-test in limma (**Supplementary Table 1**). P-values were adjusted for multiple comparisons by FDR, and denoted by *Padj < 0.05, **Padj < 0.01, ***Padj < 0.001. The association between indicated gene’s LPS response and DBP levels across all patients (or transcription factors regulating the gene) was estimated using local polynomial regression (‘loess’ function in R). The significance of all two-group comparison [such as the comparison of the LPS responses between CVD patients with normal and high blood pressure levels], as well as of regression slope were calculated by t-test, after the normality check using Shapiro-Wilk test (*P* > 0.05). All statistical analyses were performed in R (v3.6.3).

## 3 Results

### 3.1 High blood pressure in stable CAD dampens the response of monocytes to LPS

**Figure 1:**
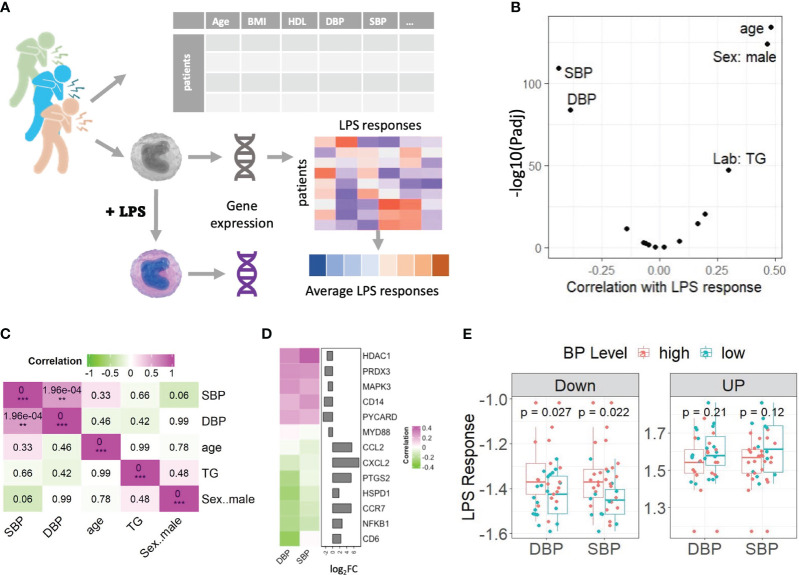
Genes’ responses to LPS were weakened in monocytes of the CAD patients with high blood pressure. **(A)** Schematic diagram of the experimental set up. **(B)** Volcano plot showing the correlation between CVD risk factors and the average LPS response signature. A positive correlation of a risk factor means the increase of this trait is associated with a stronger LPS response. **(C)** The correlations among 5 CVD risk factors based on the values from clinical records. P-values of correlation coefficients are shown in the boxes. FDR adjusted pvalue are denoted by **Padj < 0.01, ***Padj < 0.001. **(D)** Heatmap shows that the LPS responses of 13 typical genes in TLR4 pathway and their correlation with DBP and SBP are in reverse. Genes were color-coded green through to magenta to indicate value of correlation coefficients. Bar plots displayed the genes’ log_2_FCs. **(E)** Box plots compared the LPS responses of CAD patients with normal (DBP<80 or SBP<130) and high (DBP>=80 or SBP>=130) blood pressure levels, on down and up regulated genes respectively. A point on a boxplot represents the median of the most significantly up/down regulated gene’s LPS responses of a CAD patient. All correlations were calculated using Pearson’s product moment correlation coefficient. Statistical significances were calculated using Student t-test. LPS: lipopolysaccharide, BMI: Body Mass Index. HDL, High-density lipoprotein; BP, blood pressure; SBP, systolic blood pressure; DBP, diastolic blood pressure; TG, triglyceride.

In this study, we examined monocyte LPS responses in a sub cohort of stable angina pectoris patients from the CTMM – CIRCULATING CELLS Cohort of CAD patients. We only included stable angina pectoris patients in our study to mitigate CAD-independent effects arising from acute ischemia and cardiac trauma commonly observed in patients with ST-segment elevation myocardial infarction. Employing expression profiles of monocytes at both baseline and following LPS treatment in CAD patients (n=38), we initially conducted Principal Component Analysis based on the 25% highest expressed genes. This analysis aimed to explore potential associations between CVD risk factors and LPS stimulation, as illustrated in **Supplementary Figure 2**. Surprisingly, the results indicated that neither the first nor the second principal components (PC1 and PC2) could effectively discriminate between monocytes from CAD patients with or without LPS stimulation (Padj > 0.16, t test of Pearson’s correlation coefficient). Subsequently, we delved into the analysis of monocyte LPS responses, expressed as the log2 fold change (log_2_FC) between LPS-stimulated cells and baseline, seeking correlations with CVD risk factors, using the strategy depicted in **Figure 1A**. In line with previous observations that show enhanced monocyte LPS response with age (11) and male sex (12), age and sex were identified as the top two patient characteristics showing the highest positive correlation to the LPS response signature (**Figure 1B**). Unexpectedly, both systolic blood pressure (SBP) and DBP were seen to display highly significant negative correlations with the average LPS response (ρ< -0,4; *P*<10^-80^, **Figure 1B**). This to our knowledge hitherto unknown observation led us to dissect the correlation between blood pressure levels and LPS responses of monocyte, its regulation, and implications for CVD in closer detail.

First, we excluded that this correlation was reflecting an underlying association of blood pressure with the three other risk factors, that were significantly correlated with the LPS response signature (i.e., sex, age, and triglyceride level). As expected, DBP and SBP in CAD patients show significantly positive mutual correlations (**Figure 1C**). While SBP and DBP did not correlate with triglyceride levels or age, we did observe a moderate, borderline significant negative correlation (*P* = 0.06) between male sex and SBP but not DBP, indicating that systolic blood pressure of female CAD patients tended to be higher in our cohort. SBP did show a significant positive correlation with glucose levels, but no correlation was observed between glucose levels and DBP (**Supplementary Figure 1C**).

To further explore the negative correlation between the average LPS response signature and blood pressure, we selected some well-known LPS/TLR4-response genes and calculated the correlation between their LPS responses and blood pressure levels across all patients in our cohort. **Figure 1D** clearly demonstrates that the LPS-upregulated genes show negative correlations to blood pressure, while LPS-downregulated genes do the opposite. Next, we calculated the LPS response of the most significant differentially expressed genes (absolute log_2_FC >1 and Padj<0.01) for normotensive (DBP<80 mmHg, n= 20 or SBP<130, n=12) versus hypertensive CAD patients (DBP≥ 80 mmHg, n= 17 or SBP>130 mmHg, n=25) (**Figure 1E**). Clearly, patients with elevated DBP and SPB levels show a weaker LPS response (the LPS-induced downregulation and upregulation of genes is less pronounced) than those with low blood pressure, which is especially evident for downregulated genes (*P* < 0.03). Interestingly, the expression of several, though not all, LPS-induced response genes, e.g. Nuclear Factor-k-B1 (NFKB1), Cluster of Differentiation 68 (CD68), Caspase-3 (CASP3), and Heat Shock Protein Family D1 (HSPD1), already correlated with high blood pressure at baseline, suggesting their expression was already increased under non-stimulated conditions, while this association was absent after LPS stimulation (**Supplementary Figure 1E**). This might at least partly explain the dampened LPS response in patients with high blood pressure. Altogether these results suggest that high blood pressure, be it diastolic or systolic, is associated with a dampened monocyte LPS response. Since, unlike SBP, the association of DBP with other risk factors was not significant, we focused on DBP for further study.

### 3.2 An oxidative-phosphorylation-related gene module is significantly related with DBP

**Figure 2:**
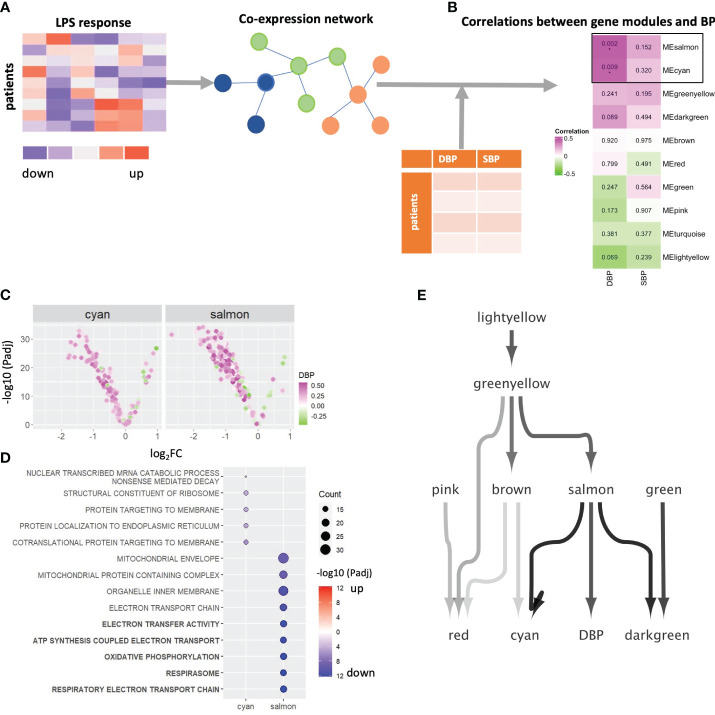
WGCNA network analysis. **(A)** Schematic diagram of the co-expression network build-up. **(B)** The correlations between eigengenes of 10 gene modules and DBP and SBP. P-values of correlation coefficients are shown in the boxes. FDR corrected pvalue are denoted by *Padj < 0.05. **(C)** Volcano plot showing each gene’s average LPS response and its association with DBP in module salmon and cyan. Genes were color-coded green through to magenta to indicate value of correlation coefficients. **(D)** A dot plot visualized the significant levels of top enriched GO terms of 2 DPB-related gene module (salmon and cyan). Significant levels were evaluated by Fisher’s exact test. Dot plots were color-coded in red (log_2_FC>0) and blue (log_2_FC < 0). Top 5 significantly enriched GO terms of module salmon were highlighted in bold. Significant levels are shown by using log10-transformed adjusted p-values. **(E)** A Bayesian network to infer the causal relations between 10 gene modules and DBP. LPS, lipopolysaccharide; SBP, systolic blood pressure; DBP, diastolic blood pressure; GO, Gene Ontology; BP, blood pressure.

To systematically map the genes that underly the strong association between LPS response in monocytes and blood pressure, we built a co-expression network for log_2_FC LPS responses of all genes, by WGCNA (25) (**Figure 2A**). Twenty gene modules (clusters) could be defined, of which modules salmon and cyan were strongly correlated with DBP (**Figure 2B**) and closely interrelated (**Supplementary Figure 3A**). Module salmon also positively correlated with LDL cholesterol levels (*P* =0.04) while module cyan showed a negative correlation (*P*=0.04) to creatinine levels, and thus kidney dysfunction (**Supplementary Figure 3B**). No modules showed significant correlations with anti-hypertensive drugs (i.e., angiotensin receptor antagonists, angiotensin-converting enzyme inhibitors, beta blocker, and calcium channel blockers, **Supplementary Figure 3B**), suggesting that the correlations are not caused by the therapy itself. To verify whether the strong association of modules salmon and cyan with DBP is confounded by other clinical parameters, we developed multiple linear regression models using limma (24), linking the eigengene of salmon and cyan with DBP, as well as with other risk factors that showed a significant correlation with the attenuated LPS response (i.e., diabetes mellitus, glucose, triglyceride, high-density and low-density lipoprotein). As shown in **Supplementary Table 1**, apart from DBP, none of these 5 additional risk factors were significantly associated with the modules salmon and cyan, thus ruling out the confounding influence of other risk factors.

As illustrated by the volcano plots in **Figure 2C** and **Supplementary Figure 3C**, most genes in modules salmon and cyan are downregulated by LPS and are positively correlated with DBP, which is not the case for other modules (**Supplementary Figure 3C**). This concurs with the earlier finding that higher blood pressure is preferentially associated with the expression of LPS downregulated genes. Over-representation analyses revealed strong enrichment of respiratory electron transport chain and oxidative phosphorylation (OXPHOS) genes in salmon (Padj < 10^-12^), while module cyan was significantly related to co-translational protein targeting to membrane (Padj = 1.09*10^-6^) and protein localization to the endoplasmic reticulum (Padj = 1.77*10^-5^, **Figure 2D**). These results suggested that higher diastolic blood pressure is associated with (LPS-induced) downregulation of genes involved in metabolic processes, particularly OXPHOS. Of note, modules red, green and greenyellow were enriched in genes related to immune responses (**Supplementary Figure 3D**).

Next, we constructed a Bayesian network model to infer the causal relation between the 10 gene modules that have at least one enriched Gene Ontology term, and DBP (**Figure 2E**). Salmon was the only module that directly connects to and impacts on DBP levels. In addition, it is also the main module able to infer cyan, the other DBP-related module. This suggests that salmon is impacting both on DBP and in parallel on cyan. In addition, we validated the link from salmon to cyan by counting the genes in downstream module cyan that were regulated by the transcription factors in the upstream module salmon in the gene regulation network. Four transcription factors from salmon were seen to regulate as many as 15 of the 76 genes contained in the cyan module, which is a highly significant enrichment (Fisher’s exact *P* = 1.4*10^-9^).

### 3.3 Gene co-expression network and regulatory network analysis on the OXPHOS-related gene module

**Figure 3:**
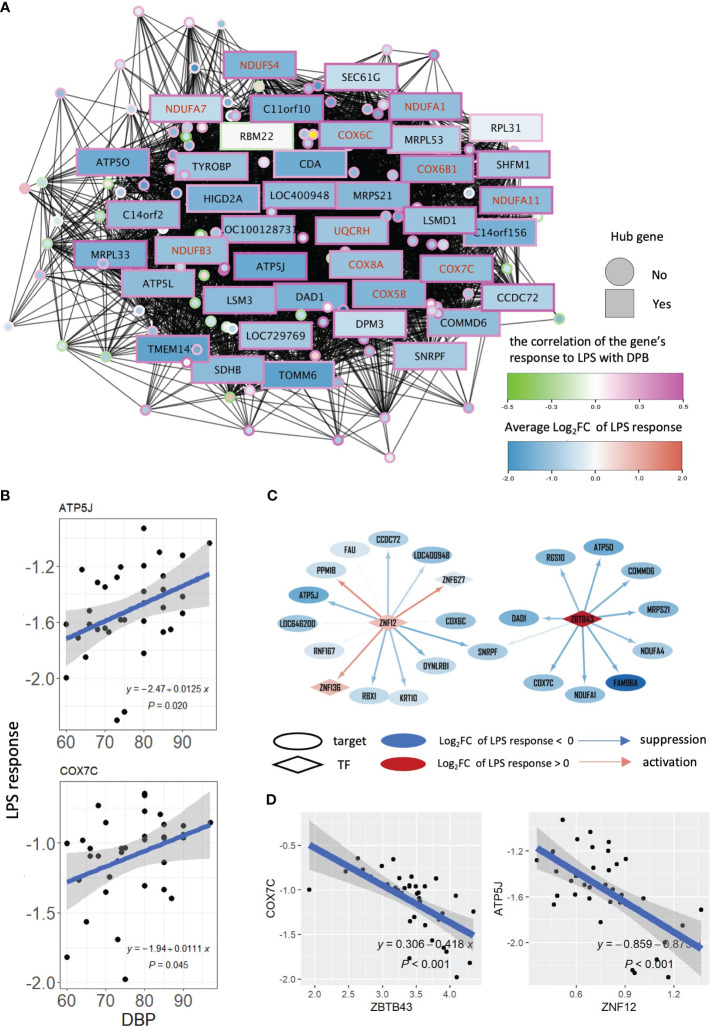
WGCNA and gene regulatory network of module salmon. **(A)** Co-expression network of module salmon. A node in the network represents a gene. The colors of node borders indicate the correlations between LPS responses and DBP. The color of a node stands for the average LPS response of this gene. Genes with red symbols are genes in the GO term: oxidative phosphorylation. **(B)** Scatters displaying the association between DBP levels and LPS response of gene ATP5J and COX7C. **(C)** Two networks showing the targets of ZNF12 and ZBTB43 in sub-gene regulatory network of module salmon. An ellipse represents a gene, and a diamond node stands for a transcription factor. A node’s color stands for the average LPS response of this gene. The color of edges of the network indicates the degree of a transcription factor repression (in blue) or activate (in red) its targets. **(D)** Association of the LPS response between ZNF12 and ATP5J, and between ZBTB43 and COX7C respectively. LPS, lipopolysaccharide; DBP, diastolic blood pressure; TF, transcription factor.

To unravel the regulatory landscape controlling module salmon and, consequently, the connection between OXPHOS in activated monocytes and DBP, we extracted the co-expression network associated with this module. Hub genes within module salmon were defined by achieving consensus among five network centrality measures (degree, betweenness, closeness, eigenvector, and page rank) using the Robust Rank Aggregation method (34) (**Figure 3A**). Consistent with earlier observations (**Figure 2C**), all but one of the 40 hub genes in module salmon were downregulated by LPS, and their responses to LPS exhibited a positive correlation with DBP (**Figure 3A**). Notably, a substantial proportion of these hub genes were identified as OXPHOS term genes, as confirmed by Fisher exact test results (*P*=7.98*10-4). These findings substantiate OXPHOS as the principal driving process within module salmon and suggest a weakened response of the module’s genes to LPS with increasing blood pressure. Subsequently, we visualized individual patients’ DBP levels along with the LPS responses of salmon’s reporter genes, including adenosine triphosphate synthase-coupling factor 6 (ATP5J, the #1 hub gene correlated with DBP) and cytochrome C oxidase subunit 7C (COX7C, a hub and OXPHOS term gene) (**Figure 3B**). The notably positive associations between DBP and the LPS responses of these genes indicate compromised monocyte OXPHOS regulation by LPS in individuals with elevated DBP.

To pinpoint the regulatory landscape of salmon, a gene regulatory network was firstly constructed using ARACNe (30) based on all genes measured in our patient cohort, and a sub-network including all target genes of the module salmon was extracted from the entire gene regulatory network (**Supplementary Figure 4A**). Based on the gene regulatory network, we inferred the transcription factor activities for each patient’s LPS response using VIPER (32), and subsequently calculated their correlations and DPB levels, to investigate which transcription factors were involved in the transcriptional response associated with changes in DBP. Zinc finger protein 12 (ZNF12) and zinc finger and BTB domain containing 43 (ZBTB43) were identified to regulate most genes in the module salmon (including COX7C and ATP5J), and their activities are significantly negatively correlated with DBP (ρ< -0.37 and *P*<0.025, **Supplementary Figure 4B**). The sub gene regulatory network of salmon in **Figure 3C** demonstrate that most of the target genes were suppressed by ZNF12 and ZBTB43. The repressive effect of these 2 transcription factors was also reflected by the negative association of ZNF12 with its target gene ATP5J, and of ZBTB43 with its target gene COX7C (**Figure 3D**). Taken together, our findings suggest that high DBP is connected to this diminished suppression.

### 3.4 In silico drug repurposing on the OXPHOS related module

**Figure 4:**
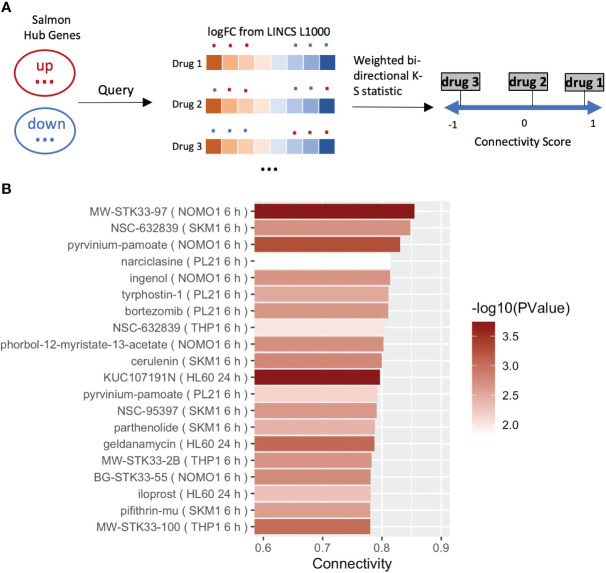
Drug reproposing based on the hub genes from module salmon. **(A)** Schematic diagram of the pursued drug repurposing pipeline. **(B)** Bar plot showing the 20 most significant drugs that can enhance the LPS responses in the result of drug reproposing. Drugs were ranked based on the Connectivity Score (or Enrichment Score). Bars of drugs were color-coded from white to red based on -log10(*P*).

Our data suggest that salmon, a module strongly correlated with DBP, is enriched in metabolic pathways/OXPHOS genes, which are downregulated by LPS. As a final step we set out to screen for drugs (by a network-guided approach), able to target the hypertension-associated module and reverse the module’s biological activity (i.e. OXPHOS) underlying the dampened LPS response in DBP and likely DBP itself. We confined to datasets obtained in myeloid-related cell lines from the LINCS L1000 repository and screened for drugs with highest ‘connectivity scores’ (36) (**Figure 4A**). Only drugs with high statistical significance (Padj <0.05) and high connectivity score (>0.7) were considered effective. Consistent with previously reported association of single nucleotide polymorphisms (SNPs) in the serine-threonine kinase STK33 gene with hypertension in Europeans (37), our investigation revealed several STK33 inhibitors as effective drugs targeting the attenuated LPS response associated with hypertension (**Figure 4B**). Notably, the top candidate drug, MW-STK33-97, was previously identified by us as one of the leading compounds capable of neutralizing a detrimental gene program in macrophages exposed to the macroenvironment of acute myocardial infarction patients (38). Our findings add to this notion and suggest that MW-STK33-97 may enhance monocyte responses to LPS, concomitantly downregulating genes involved in OXPHOS.

## 4 Discussion

In this study, we examined the impact of CVD risk factors on monocyte inflammatory response capacity in a cohort of stable coronary artery disease patients. To our surprise, we found a negative correlation between blood pressure and monocyte LPS-responses, suggesting dampened LPS response capacity in patients with high blood pressure. WGCNA analysis identified a co-expressed gene module (salmon) to correlate with DBP. This module was enriched in OXPHOS and respiratory electron transport chain term genes. While most of its hub genes were downregulated by LPS, this suppression was significantly weaker in patients with high DBP.

As inferred from Bayesian network analysis module salmon is upstream of DBP, suggesting that this module, enriched in OXPHOS genes, directly or indirectly impacts on DBP. This finding was not confounded by hidden correlations between DBP and other risk factors such as age, gender, or cholesterol levels. Although SBP showed a positive correlation to glucose levels and diabetes mellitus, this was not the case for DBP. Moreover, the correlation between DBP and module salmon was maintained even despite antihypertensive treatment. Monocyte/macrophage activation is known to induce pro-inflammatory responses, which are fostered by a shift in metabolism towards enhanced glycolysis and reduced OXPHOS (39). A dampened metabolic response may compromise the inflammatory response, as has been reported before (40).

A direct link between monocyte OXPHOS and hypertension is to our knowledge hitherto unknown. The observed link could implicate monocyte metabolic dysfunction in the development of DBP, but it may well reflect systemic metabolic dysfunction, including in cell types known to control DBP, such as the endothelial and smooth muscle cells of the microvasculature. Indeed, impaired mitochondrial activity (OXPHOS) has been repeatedly shown to mark many (age-related) cardiometabolic diseases and to induce microvascular dysfunction in cardiomyopathy, as well as in portal (41), pre-eclampsic (42), and pulmonary hypertension (43) (for a review see (44):). LPS-induced pro-inflammatory responses are known to be associated with mitochondrial dysfunction and excessive formation of reactive oxygen species, factors that also can induce vascular dysfunction, hypertension and atherosclerosis (17, 45). Vice versa, accumulating evidence suggests that hypertension itself can increase oxidative stress in vasculature and heart, leading to a vicious cycle mitigating disease (2). Alternatively, the dampened monocyte response may act as a proxy for a broader (endothelial/microcirculatory) dampening of LPS response capacity in hypertension, rather than being a direct causal factor in hypertension. Whether the observed co-expressed monocyte module is a mere indicator of, or a causal factor in DBP, e.g. possibly by trained immunity (22), remains subject for further study.

Alternatively, the dampened response could also be due to low grade chronic inflammation, often observed in hypertensive patients (46, 47). In fact, (low grade) inflammation/pro-inflammatory cytokines have been shown to activate the renin-angiotensin-aldosterone system, even in local vascular tissue, leading to endothelial dysfunction and vascular stiffening (48, 49). In turn, high blood pressure can cause low grade inflammation itself (48). The elevated baseline expression (i.e. without LPS stimulation) of several TLR4 response genes, such as NFKB1, CD68, CASP3 and HSPD1, in hypertensive patients, corroborates this notion and could at least partly explain why responses to the subsequent LPS stimulation were dampened. It should be noted though, that C-reactive protein levels were not correlated with DBP in our cohort (*P*=0.41), making it less probable that the dampened inflammation is caused by DBP-associated low-grade inflammation.

The main hub genes in the module salmon are well known for their role in OXPHOS. COX7C is part of the terminal component of the mitochondrial respiratory chain, while ATP5J catalyzes ATP synthesis during OXPHOS. Interestingly, ATP5J overexpression in mice on a high salt diet led to increased blood pressure levels (50). For COX7C no direct link to hypertension has been demonstrated so far, though COX7C expression was increased in PBMCs of chronic kidney disease patients, which often display hypertension (51). Considering the transcriptional regulation of the salmon co-expression network, we identified ZBTB43 and ZNF12 as transcription factors that were most extensively connected to network members (including COX7C and ATP5J) and their activities are most relevant to DBP. Interestingly, ZBTB43 has been identified as a quantitative trait locus (QTL) associated with high blood pressure, as well as familial hyperlipidemia (https://rgd.mcw.edu/rgdweb/report/gene/main.html?id=1310287#pubMedReferences), which is in line with our findings that module salmon is not only associated with DPB but also to some extent with LDL cholesterol levels. Similarly, ZNF12, the regulator of ATP5J as well as COX6C, was also identified as hypertension-associated QTL in rats (52).

While these findings provide circumstantial evidence, they point to a direct connection between (LPS-induced suppression of) OXPHOS and elevated blood pressure. This notion gains further support from our subsequent drug repurposing screening, revealing several candidates associated with blood pressure regulation or hypertension to target the core of the identified module. In addition to STK33 inhibitors, the broad-spectrum deubiquitinating enzyme inhibitor NSC-632839 and pyrvinium pamoate emerged as top candidates with the highest connectivity scores. Intriguingly, the ubiquitin proteasome system, targeted by NSC-632839, has already been recognized for its crucial role in blood pressure regulation (53). Pyrvinium, a Wnt/beta-catenin inhibitor, has demonstrated cardioprotective effects by improving calcium homeostasis and mitigating mitochondrial dysfunction (54). Notably, emerging evidence suggests a role for Wnt signaling in the regulation of blood pressure (55–57). It is noteworthy that even apparently healthy individuals exhibit detectable levels of endotoxin in plasma (18). This may be derived from gram-negative bacteria that colonize the gastrointestinal, genitourinary, and respiratory tracts amongst others. Endotoxin levels may be elevated not only during infections but also in common subclinical or chronic conditions like periodontitis, sinusitis, or bronchitis. Interestingly, increased intestinal barrier disruption, often observed in patients and mouse models of CVD, is accompanied by a concurrent elevation in plasma endotoxin levels, inevitably contributing to systemic inflammation, hypertension, and atherosclerosis (58). Collectively, this endorses the (patho)physiological relevance of our experimental setting and findings.

A limitation of this study lies in the relatively modest number of participants and its exclusive focus on patients with clinically manifest CAD, presenting a complex constellation of cardiovascular disease risk factors. Due to the deliberate inclusion of only CAD patients in our cohort, the study design did not allow examination of the module’s correlation with hypertension in a non-CAD context. Furthermore, we acknowledge that a significant proportion of CAD patients in our cohort had undergone anti-hypertensive treatment, often in conjunction with lipid-lowering or anticoagulant drugs. While we successfully ruled out a confounding role of drug treatment on the module’s correlation with DBP for various medications, including angiotensin-converting enzyme inhibitors, beta-blockers, angiotensin receptor antagonists, and calcium channel blockers (both individually and collectively), the presence of medication may still complicate the data interpretation. The observed correlation in hypertensive patients undergoing anti-hypertensive treatment, however, suggests that the module exposes a common underlying disease mechanism that is either not targeted or inadequately addressed by the prescribed antihypertensive drugs themselves. Finally, monocyte LPS responsiveness was studied *ex vivo*. Although the isolation procedure was performed on ice, LPS stimulation was short (15 min), precluding early differentiation effects, and baseline monocytes did not show any indication of activation because of sample preparation and bead isolation, we cannot exclude that monocytes ex vivo behave different than *in vivo*.

In conclusion, our study demonstrated a strong negative association of diastolic blood pressure with LPS responses in monocytes of advanced, stable CAD patients, and identified a gene cluster sharply enriched in OXPHOS term members to mediate this correlation. Drug repurposing screening showed that serine-threonine inhibitor MW-STK33-97 could be able to reverse this DBP-associated gene profile (or target this disease cluster). While pointing towards a causal relationship between diastolic blood pressure and OXPHOS in activated monocytes, the actual direction of the observed relationship remains to be determined.

## Data availability statement

The datasets presented in this study can be found in online repositories. The names of the repository/repositories and accession number(s) can be found below: https://www.ncbi.nlm.nih.gov/, GSE234398. Processed transcriptomics data and patients’ information are available at https://github.com/ChangLu92/LPSStudy/tree/main/data. All codes used for analysis are available at https://github.com/ChangLu92/LPSStudy. For the raw baseline data, please contact luchang4104@gmail.com.

## Ethics statement

The studies involving humans were approved by the Institutional Medical Ethical Review Board of the University Medical Center Utrecht, The Netherlands. The studies were conducted in accordance with the local legislation and institutional requirements. The participants provided their written informed consent to participate in this study. Written informed consent was obtained from the individual(s) for the publication of any potentially identifiable images or data included in this article.

## Author contributions

CL: Methodology, Software, Visualization, Writing – original draft, Writing – review & editing, Data curation, Funding acquisition, Project administration, Formal analysis. MD: Conceptualization, Project administration, Supervision, Writing – original draft, Writing – review & editing. JB: Data curation, Software, Writing – review & editing. HJ: Data curation, Methodology, Writing – review & editing. JO: Data curation, Investigation, Project administration, Writing – review & editing. MM: Investigation, Project administration, Writing – review & editing. AZ: Resources, Writing – review & editing, Validation. JJ: Resources, Writing – review & editing. AK: Resources, Writing – review & editing. JK: Resources, Writing – review & editing. GP: Resources, Writing – review & editing. BM: Resources, Writing – review & editing. JS: Supervision, Writing – review & editing. RC: Methodology, Supervision, Writing – review & editing. JMK: Methodology, Supervision, Writing – review & editing. PG: Supervision, Writing – review & editing. EB: Conceptualization, Supervision, Writing – review & editing, Funding acquisition, Project administration.
