# Supplementary material for: Identification of a gene network driving the attenuated response to lipopolysaccharide of monocytes from hypertensive coronary artery disease patients
Source: Front Immunol. 2024 Feb 12;15:1286382. doi: 10.3389/fimmu.2024.1286382 (PMC10894924; doi:10.3389/fimmu.2024.1286382)
Supplement: Supplementary file 1 [file DataSheet_1.docx]

Supplementary Material

A gene network driving hypertension associated dampened monocyte LPS response in coronary artery patients

**Chang Lu, Marjo M.P.C. Donners*, Julius B. J. de Baaij, Han Jin, Jeroen J.T. Otten, Marco Manca, Anton Jan van Zonneveld, J. Wouter Jukema, Adriaan Kraaijeveld, Johan Kuiper, Gerard Pasterkamp, Barend Mees, Judith C. Sluimer, Rachel Cavill, Joël M.H. Karel, Pieter Goossens, Erik A.L. Biessen**

*** Correspondence:** Dr. Marjo Donners: [marjo.donners@maastrichtuniversity.nl](mailto:marjo.donners@maastrichtuniversity.nl)

# Supplementary Figures


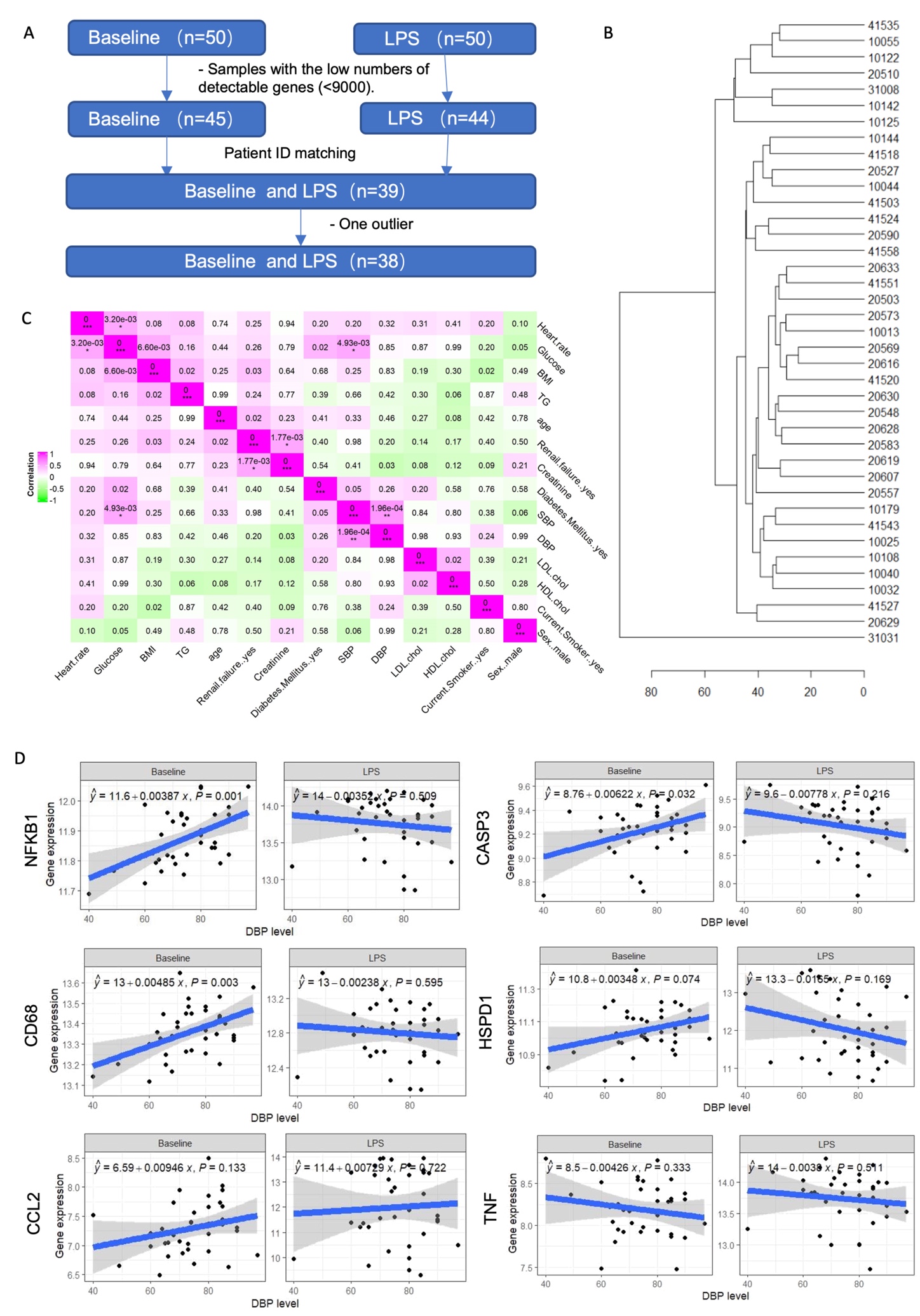


**Supplementary Figure 1.** **Cohort build-up, data preprocessing, and correlation analysis.** (A) Schematic diagram of the cohort build-up. (B) Hierarchy tree showing the similarities among 39 CAD samples based on the LPS response matrix. (C) The correlations among 14 risk factors are based on the clinical data. All correlations were calculated using Pearson's product moment correlation coefficient. Statistical significances were calculated using Student t-test. P-values are shown in the boxes. FDR adjusted pvalue are denoted by *Padj < 0.05, **Padj < 0.01, ***Padj < 0.001. (D) Scatters showing the association between DBP levels and the expression of 6 LPS responsive genes in the LPS-TLR4 pathway on Baseline and LPS-stimulated microarray data. LPS: lipopolysaccharide, HDL: high-density lipoprotein, LDL: low-density lipoprotein, SBP: systolic blood pressure, DBP: diastolic blood pressure, TG: triglyceride.


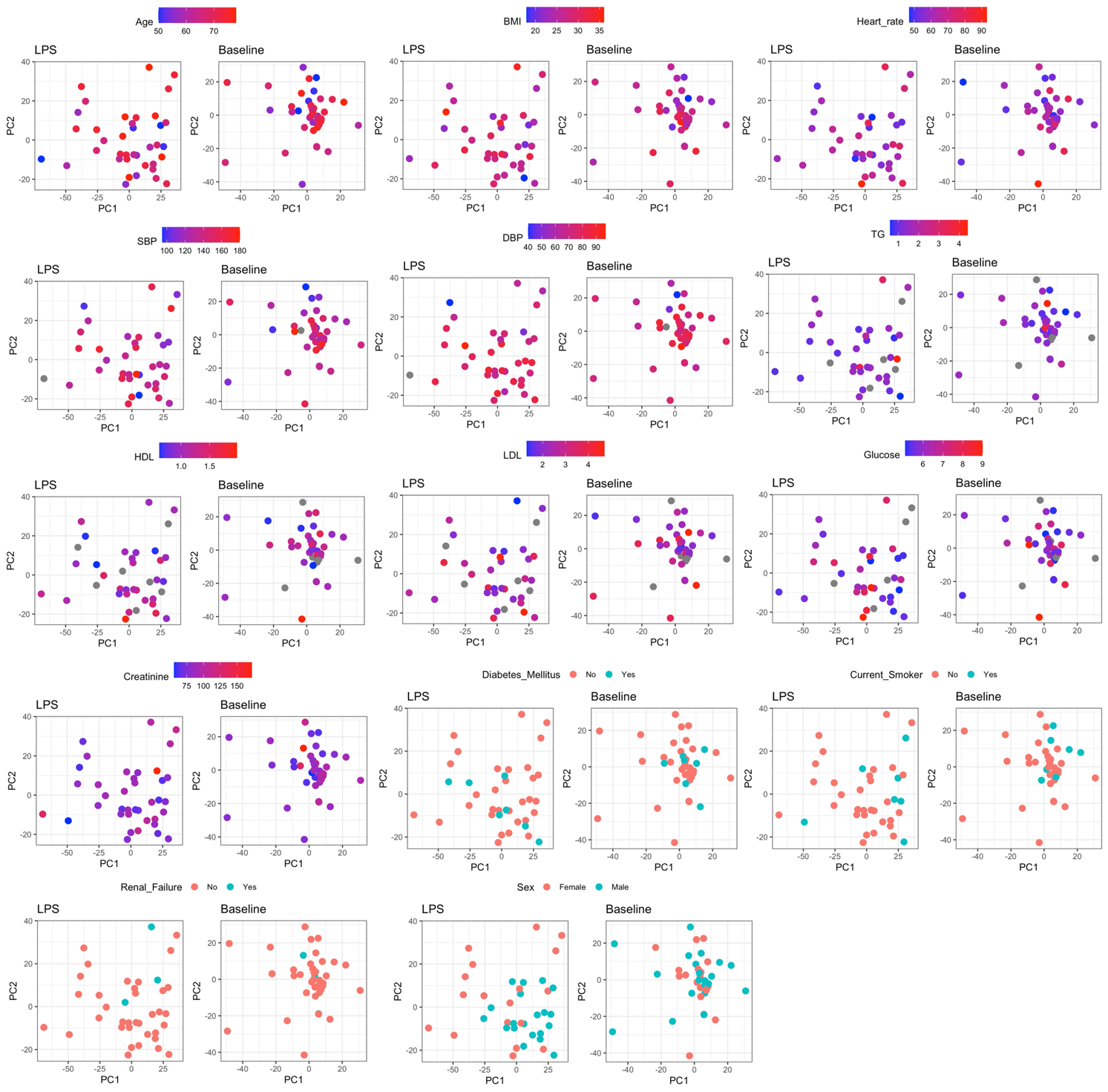


**Supplementary Figure 2.** **PCA analysis based on the 25% highest expressed genes from baseline and LPS-stimulated samples differentiating for 14 CVD risk factors.** LPS: lipopolysaccharide, HDL: high-density lipoprotein, LDL: low-density lipoprotein, SBP: systolic blood pressure, DBP: diastolic blood pressure, TG: triglyceride.


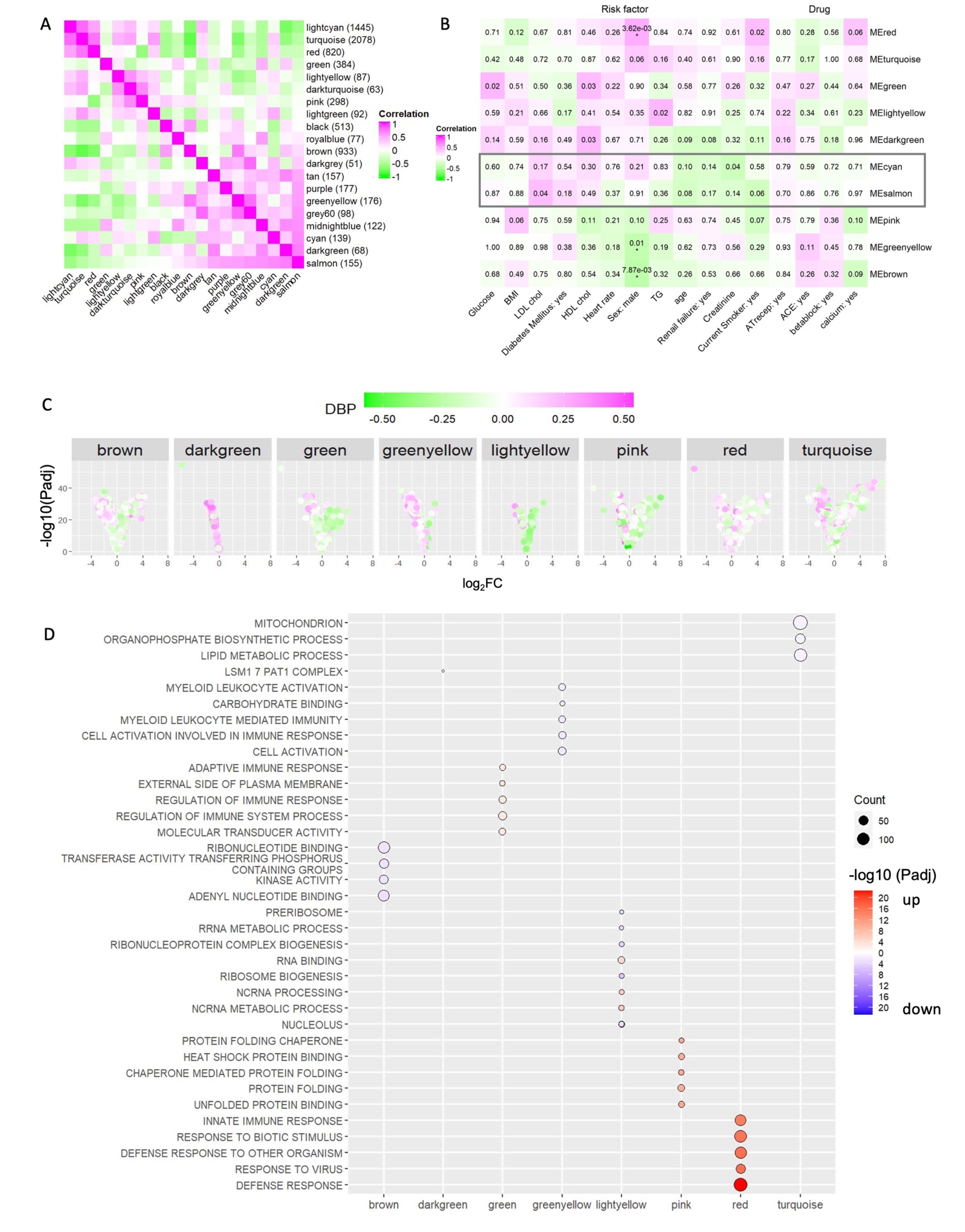


**Supplementary Figure 3.** **WGCNA analysis based on the LPS response matrix.** (A) A heatmap showing the similarities (correlation) among the gene modules. The numbers in the parentheses indicate the number of genes per module. (B) The correlations between eigengenes with CVD risk factors and anti-hypertension drugs. Heatmap was color-coded green through to magenta to indicate the direction of correlation coefficients. Statistical significances were calculated using Student t-test. P-values of correlation coefficients are shown in the boxes. FDR corrected pvalue are denoted by *Padj < 0.05, **Padj < 0.01, ***Padj < 0.001. (C) Volcano plot showing each gene’s average LPS response and its association with DBP 8 of 10 modules (except for salmon and cyan). (D) Dot plot visualizing the top 5 enriched GO terms involved in the molecular function, biological process, and the cellular component. Dots were color-coded in red (log_2_FC>0) and blue (log_2_FC < 0). Significant levels are shown by using log10-transformed adjusted p-values. GO: Gene Ontology, LPS: lipopolysaccharide, HDL: high-density lipoprotein, LDL: low-density lipoprotein, SBP: systolic blood pressure, DBP: diastolic blood pressure, TG: triglyceride, ACE: angiotensin-converting enzyme.


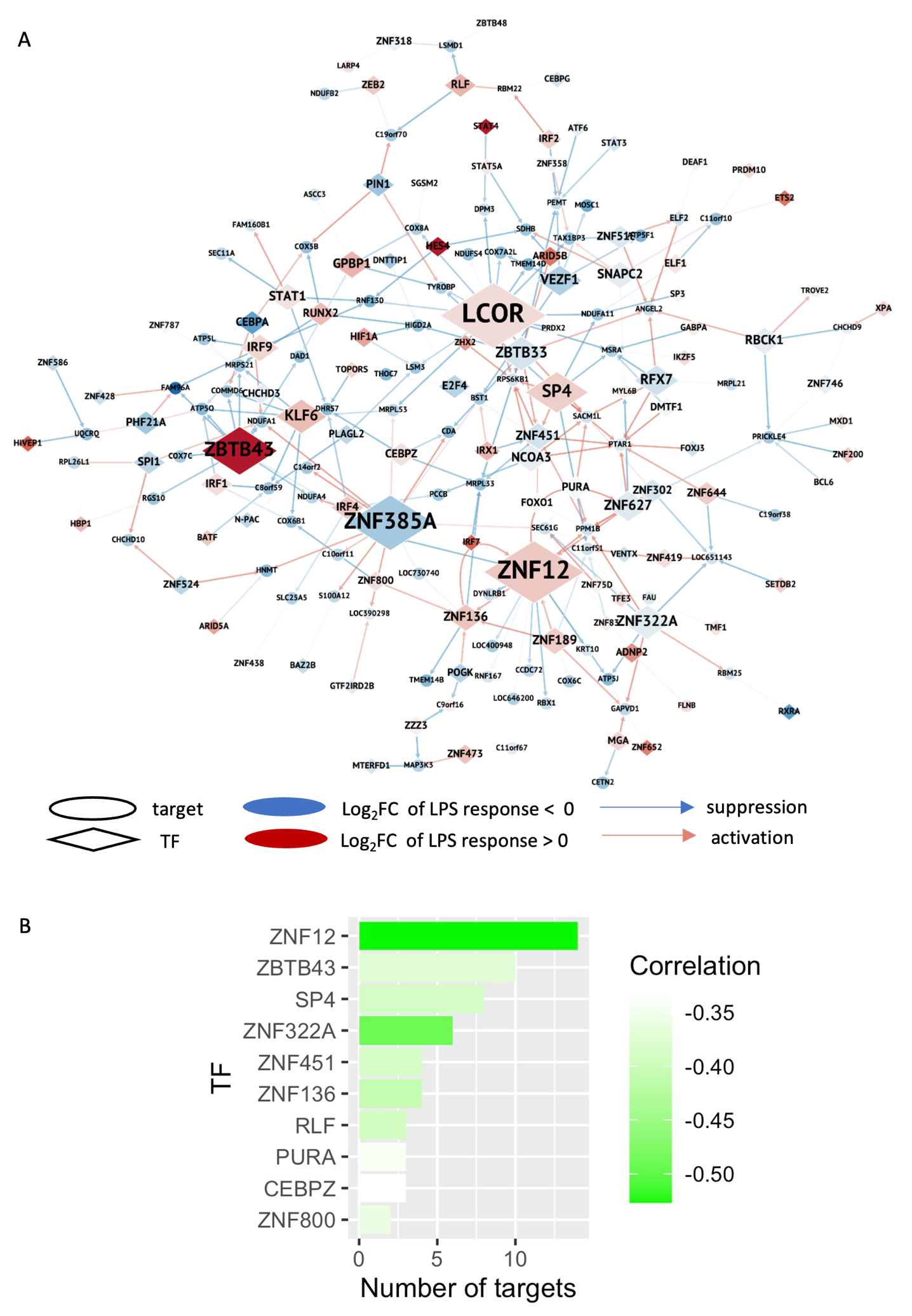


**Supplementary Figure 4.** **Gene regulatory network of module salmon and** transcription factor **activity inference.** (A) Gene regulatory subnetworks of module salmon. An ellipse represents a gene, and a diamond node stands for a transcription factor. A node’s color represents the average LPS response of this gene. The color of the edges of the network indicates the degree to of a transcription factor represses (in blue) or activates (in red) its targets. The size of nodes is positively associated with the number of targets that transcription factors regulated in module salmon. The names of transcription factors were shown on the diamond nodes. (B) Bar plot showing the top transcription factors that significantly correlated to DBP levels (*P*<0.05). The x-axis indicates the number of targets of a transcription factor, and the y-axis represents transcription factor names. Bars are color-coded by the correlations between transcription factor activities and DBP levels. LPS: lipopolysaccharide, DBP: diastolic blood pressure, TF: transcription factor.

# Supplementary Table

**Supplementary Table 1:** **P-values for DBP as well as other clinical parameters showing significant negative correlation to LPS response in the multiple linear regression model.** Linear modeling of WGCNA Eigengenes and DBP was performed using limma, with diabetes, glucose, triglyceride, HDL, and LDL as covariates (see Materials and Methods 2.13). HDL: high-density lipoprotein, LDL: low-density lipoprotein, DBP: diastolic blood pressure.

| Module | DBP | Diabetes | Glucose | Triglyceride | HDL | LDL |
| --- | --- | --- | --- | --- | --- | --- |
| **salmon** | 0,014 | 0,702 | 0,945 | 0,947 | 0,947 | 0,114 |
| **cyan** | 0,008 | 0,441 | 0,177 | 0,140 | 0,186 | 0,422 |
